# Demographic, Health and Lifestyle Factors Associated with the Metabolome in Older Women

**DOI:** 10.3390/metabo13040514

**Published:** 2023-04-03

**Authors:** Sandi L. Navarro, G. A. Nagana Gowda, Lisa F. Bettcher, Robert Pepin, Natalie Nguyen, Mathew Ellenberger, Cheng Zheng, Lesley F. Tinker, Ross L. Prentice, Ying Huang, Tao Yang, Fred K. Tabung, Queenie Chan, Ruey Leng Loo, Simin Liu, Jean Wactawski-Wende, Johanna W. Lampe, Marian L. Neuhouser, Daniel Raftery

**Affiliations:** 1 Cancer Prevention Program, Division of Public Health Sciences, Fred Hutchinson Cancer Center, Seattle, WA 98109, USA; ltinker@whi.org (L.F.T.); rprentic@whi.org (R.L.P.); jlampe@fredhutch.org (J.W.L.); mneuhous@fredhutch.org (M.L.N.); 2 Department of Anesthesiology and Pain Medicine, University of Washington, Seattle, WA 98109, USA; ngowda@uw.edu (G.A.N.G.); lisafanbettcher@gmail.com (L.F.B.); rpepin@iu.edu (R.P.); natalienguyen1110@gmail.com (N.N.); mellenberger20@gmail.com (M.E.); 3 Department of Biostatistics, University of Nebraska Medical Center, Omaha, NE 68198, USA; cheng.zheng@unmc.edu; 4 Biostatistics Program, Division of Public Health Sciences, Fred Hutchinson Cancer Center, Seattle, WA 98109, USA; yhuang@fredhutch.org; 5 School of Public Health, Xinjiang Medical University, Urumqi 830011, China; tyang23@fredhutch.org; 6 Department of Internal Medicine, Division of Medical Oncology, College of Medicine and Comprehensive Cancer Center, The Ohio State University, Columbus, OH 43210, USA; fred.tabung@osumc.edu; 7 School of Public Health, Imperial College of London, London SW7 2AZ, UK; q.chan@imperial.ac.uk; 8 Australian National Phenome Centre, Health Futures Institute, Murdoch University, Murdoch, WA 6150, Australia; rueyleng.loo@murdoch.edu.au; 9 Center for Global Cardiometabolic Health, Department of Epidemiology, School of Public Health, Providence, RI 02912, USA; simin_liu@brown.edu; 10 Department of Medicine and Surgery, Alpert School of Medicine, Brown University, Providence, RI 02903, USA; 11 Department of Epidemiology and Environmental Health, University at Buffalo, Buffalo, NY 14214, USA; jww@buffalo.edu

**Keywords:** metabolomics, confounders, correlates, NMR, mass spectrometry

## Abstract

Demographic and clinical factors influence the metabolome. The discovery and validation of disease biomarkers are often challenged by potential confounding effects from such factors. To address this challenge, we investigated the magnitude of the correlation between serum and urine metabolites and demographic and clinical parameters in a well-characterized observational cohort of 444 post-menopausal women participating in the Women’s Health Initiative (WHI). Using LC-MS and lipidomics, we measured 157 aqueous metabolites and 756 lipid species across 13 lipid classes in serum, along with 195 metabolites detected by GC-MS and NMR in urine and evaluated their correlations with 29 potential disease risk factors, including demographic, dietary and lifestyle factors, and medication use. After controlling for multiple testing (FDR < 0.01), we found that log-transformed metabolites were mainly associated with age, BMI, alcohol intake, race, sample storage time (urine only), and dietary supplement use. Statistically significant correlations were in the absolute range of 0.2–0.6, with the majority falling below 0.4. Incorporation of important potential confounding factors in metabolite and disease association analyses may lead to improved statistical power as well as reduced false discovery rates in a variety of data analysis settings.

## 1. Introduction

The field of metabolomics involves the parallel measurement of large numbers of small molecules in biological systems and offers new avenues for understanding biological phenotypes, deciphering mechanisms, and identifying biomarkers or drug targets for a variety of diseases [1,2,3,4,5,6]. The currently detectable metabolome is complex and rich; tens of thousands of endogenous human body metabolites have been identified thus far, with a majority belonging to various lipid classes. These metabolites represent the downstream products of gene expression and protein action and thus provide an instantaneous snapshot of biological phenotype. To date, metabolomics studies have resulted in numerous important findings in systems biology and biomarker discovery, including a deeper understanding of the relationship between metabolism and chronic diseases [7]. Furthermore, these findings have the potential to improve early disease detection or therapy monitoring, as well as application to research areas such as environmental and nutritional sciences.

Metabolite profiling of biological specimens such as biofluids and tissues is primarily based on two analytical platforms: nuclear magnetic resonance (NMR) spectroscopy [8,9,10,11,12,13,14,15,16] and mass spectrometry (MS) [17,18,19,20,21,22,23]. NMR’s excellent reproducibility (coefficient of variation, CV ~1–3%) and its high quantitative accuracy, as well as its ability to identify structures of unknown metabolites, offer unique strengths for metabolomics applications. MS, including gas chromatography (GC)- and liquid chromatography (LC)-MS, on the other hand, owing to its inherently high sensitivity, plays a dominant role in the metabolomics field.

In the area of diet and nutrition studies, metabolomics plays a key role in the development of nutritional biomarkers (e.g., objective measure of nutrient or food intake) to better understand the relationship between dietary intake and chronic disease risk [24]. To date, most diet-disease association studies depend on observational data. A challenge, however, is that despite a broad range of available metabolomics technologies that now routinely enable the identification of dietary intake serum or urine biomarkers, the deleterious effect of confounding factors on such biomarker candidates is a major bottleneck [25]. It is becoming increasingly apparent that metabolite profiles are influenced by numerous clinical and demographic factors, such as sex, age, body mass index (BMI), smoking, alcohol consumption, and medication use, such as lipid-lowering and anti-inflammatory drugs [2,26,27,28]. Consequently, the derived biomarkers can be confounded by one or more of these factors [2,29,30,31,32,33].

To address this challenge, we investigated the correlation of more than 1000 serum and urine metabolites, including lipids, with numerous demographic, lifestyle, and clinical parameters in a subset of a racially and ethnically diverse, well-characterized observational cohort of 444 post-menopausal women in the Women’s Health Initiative (WHI), for which extensive diet and clinical data have been collected. Our overarching goal was to determine which factors are most important for adjustment when investigating metabolites in disease association studies.

## 2. Materials and Methods

### 2.1. Overview of the Women’s Health Initiative Observational Study

The Women’s Health Initiative (WHI) Observational Study (WHI-OS) is a prospective study of 93,676 postmenopausal women who were enrolled between 1994 and 1998 at 40 clinical centers in the United States. Details on study design and recruitment have been published previously [34,35,36]. Data and biological samples for this present analysis were derived from the Nutrition and Physical Activity Assessment Study Observational Study (NPAAS-OS), an ancillary study derived from the WHI, conducted at 9 WHI clinical centers between 2006 and 2009 [37]. The NPAAS study was approved by the institutional review board, and all women gave informed written consent, in addition to ongoing consent for WHI participation. The WHI is registered at clinicaltrials.gov (NCT00000611).

### 2.2. Study Population

Details about the study population have been published [37]. A total of 450 WHI-OS participants completed a fasting blood draw, a 24 h urine collection, anthropometry, and 3 dietary assessment tools: a food frequency questionnaire (FFQ), a 4-day food record, and 3 24 h recalls. In this analysis, only the FFQ data were used. Most of these activities were carried out during 2 clinic visits over a period of 2 weeks. Blood samples were collected in the fasting state (≥12 h) and were maintained at 4 °C for up to 1 h until serum was separated from cells. Centrifuged aliquots were stored in freezers (at −80 °C) until analysis [38] For urine collections, participants were instructed to begin collections after a first-morning void preceding the penultimate study day and to record any missed voids or spillage. Boric acid was used as a preservative. [39]. Data collected at the NPAAS-OS clinic visit included participant’s age, height, and weight, and reporting of current cigarette smoking, medication use (duration and frequency), dietary supplement use (type and daily average amount), [40] and physical activity using standardized WHI instruments. Race and ethnicity (self-identified), height, baseline income, education, and marital status were obtained from the primary WHI database at baseline enrollment 1993–1998. Medication use was obtained from data collected at the end of the first WHI extension study in 2010. A thorough medication inventory system was developed to capture the use of all usual medications, including formulation, dose, and duration, and whether these medications were prescription or over-the-counter drugs [34]. A total of 1653 different medications were reported among 212 women in NPAAS-OS. Any medication class used by >5% of women were retained for analysis. Self-reported recreational physical activity was categorized as metabolic equivalent task (MET) hours, where METs are used to specify intensity, e.g., light, moderate, or vigorous. Total recreational physical activity is then calculated as MET hours/week by multiplying individual exercise METs by hours per week engaged in that activity and summing over all types of activities. NPAAS-OS recruitment was oversampled for participants with extremes of body mass index (BMI; <18.5 and ≥30.0 kg/m^2^), women aged ≤59 years at WHI enrollment, and self-identified Black and Hispanic/Latina women. Exclusion criteria included medical conditions precluding participation, weight instability, or travel plans during the study period. Participants were instructed to follow their usual diet, dietary supplement use, and physical activity habits during the study. A total of 444 women for whom serum and urine samples were available were included in this present analysis.

### 2.3. Metabolomics Analysis

#### 2.3.1. Serum

Details on metabolite measurements have been published previously [41]. Briefly, for the analysis of aqueous blood metabolites, methanol extracts of fasting serum samples from 444 NPAAS-OS participants, along with 17 blinded duplicates were analyzed by targeted LC-MS/MS using an AB Sciex Triple Quad 6500+ mass spectrometer [42]. The LC system was composed of four Shimadzu Nexera LC-20 pumps, an AB Sciex/CTC autosampler, and Agilent 1260 column compartment containing a column-switching valve integral to the MS instrument (Agilent Instrument Technologies, Santa Barbara, CA, USA). A total of 2 HILIC (hydrophilic interaction chromatography) columns (Waters XBridge Amide; 150 × 2.1 mm, 2.5 µm particle size), connected in parallel, were used for positive and negative ionization modes. Each sample was injected twice, 10 μL for analysis using negative ionization mode and 5 μL for analysis using positive ionization mode. Our setup allows one column to perform the separation, while the other column is reconditioned and readied for the next injection. The flow rate was 0.300 mL/min, the autosampler temperature was kept at 4 °C, the column compartment was set at 40 °C, and the total separation time for both ionization modes was 20 min. The mobile phase was composed of Solvents A (5 mM ammonium acetate in 88% H_2_O, 10% acetonitrile, 2% methanol +0.2% acetic acid) and B (5 mM ammonium acetate in 88% acetonitrile, 10% H_2_O, 2% methanol + 0.2% acetic acid). Identical gradient conditions were used for both separations. The assay was developed using authentic commercially obtained compounds (Sigma-Aldrich, Saint Louis, MO, USA or Fisher Scientific, Pittsburgh, PA, USA) and targeted a total of 303 metabolites that represent >40 different metabolic pathways, along with 33 stable isotope labeled internal standards (Cambridge Isotope Laboratory, Tewksbury, MA, USA). Metabolite concentrations were obtained using MultiQuant 3.0.2 software. Of the 303 metabolites targeted, 157 were detected in >80% of all samples.

Separately, quantitative analysis of serum lipids was performed using the Lipidyzer^TM^ platform consisting of Shimadzu (Shimadzu, Columbia, MD, USA) Nexera X2 LC-30AD pumps, a Shimadzu Nexera X2 SIL-30AC autosampler, and an AB Sciex Q TRAP^®^ 5500 mass spectrometer (Sciex, Framingham, MA, USA) equipped with SelexION^®^ for differential mobility spectrometry (DMS) [43,44]. The method used 1-propanol as the chemical modifier for the DMS. Lipid metabolites were first extracted using dichloromethane/methanol to isolate the lipid species and remove proteins. The method targeted 1070 different lipids representing 13 different lipid classes as follows: phosphatidylcholine (PC), phosphatidylethanolamine (PE), lysophosphatidylcholine (LPC), lysophosphatidylethanolomine (LPE), sphingomyelin (SM), cholesterol ester (CE), ceramide (CER), diacylglycerol (DAG), dihydroceramide (DCER), free fatty acid (FFA), hexosylceramide (HCER), lactosylceramide (LCER), and triacylglycerol (TAG) classes of lipids. Data were acquired and processed using Analyst 1.6.3 and Lipidomics Workflow Manager 1.0.5.0. Absolute concentrations (in µM) were obtained based on a mixture of 54 isotope labeled internal standards, which were added to each sample during sample preparation. In total, this approach resulted in 756 serum lipids detected in >80% of all samples.

#### 2.3.2. Urine

Metabolite profiles for 24 h urine samples (blinded duplicates) were analyzed by ^1^H 800 MHz NMR spectroscopy. For NMR analysis, urine samples (300 µL each) were mixed with an equal volume of phosphate buffer (100 mM, pH = 7.4) containing an internal standard, TSP (3-(trimethylsilyl)propionic-2,2,3,3-d_4_ acid sodium salt, 25 µM), and transferred to 5 mm NMR tubes. The samples were analyzed using a Bruker Avance II 800 MHz NMR spectrometer equipped with a cryogenically cooled probe and Z-gradients suitable for inverse detection. One-dimensional NMR experiments using the ‘noesyprld’ pulse sequence with water suppression using presaturation were performed under identical experimental conditions. Each spectrum was obtained using 10,000 Hz spectral width and 32,768 time-domain data points. Free induction decay (FID) signals were Fourier transformed after multiplying using an exponential window function and a line broadening of 0.5 Hz after setting the spectrum size to 32,768 points. Resulting spectra were phase and baseline corrected, and the chemical shifts were referenced to the internal TSP peak. Metabolites were then identified based on the literature and chemical shift databases [45,46]. Metabolite concentrations were obtained after normalizing NMR spectra with reference to the internal standard, TSP, peak. Bruker Top-Spin versions 3.0 and 3.1 software packages were used for NMR data acquisition and processing, and Bruker AMIX software was used for metabolite quantitation. Relative spectral abundances for 58 metabolites were obtained. None of the metabolites had any missing values.

Separately, urine samples were analyzed using global GC-MS using an Agilent 7890/5975 GC-MS instrument and following established protocols [18]. Urine samples were treated with urease enzyme to deplete the urea level followed by methoxymation using methoxime. Urine metabolites were then derivatized using MSTFA (N-Methyl-N-(trimethylsilyl) trifluoroacetamide) with 1% (*v*/*v*) TMSC (trimethylchlorosilane). Prior to derivatization, the samples were mixed with myristic acid-d_27_ and a FAME (fatty acid methyl-ester) mixture of retention time index compounds. The samples (1 µL) were injected onto the instrument using splitless mode. Helium was used as the carrier gas with a flow rate of 1.2 mL/min. Separation was performed using an Agilent DB-5 ms + 10 m Duraguard capillary column (20 m × 250 µm × 0.25 µm). The column temperature was maintained at 60 °C for 1 min, then increased at a rate of 10 °C/min to 325 °C and held at this temperature for 10 min. Mass spectral signals were collected after a solvent delay of 4.90 min. Peak intensities and elution times for the retention time index compounds were verified by *m/z* values after each experiment. After converting the data to the appropriate format, MS peaks were analyzed using Agilent MassHunter Quantitative Analysis software and PARADISe version 1.1.6 [47]. Relative concentrations of metabolites were obtained after normalizing the data with respect to the internal standard, myristic acid-d_27_. This resulted in a total of 275 metabolites, 137 of which were named compounds, observed in >80% of all samples.

### 2.4. Quality Controls (QC) Used in the Metabolite Analysis

Analysis protocols used multiple layers of QC samples as well as isotope labeled or unlabeled internal standards to assess instrument stability/performance during the analysis and help with normalization and metabolite quantitation. Different types of QCs used included: (a) unblinded instrument QC samples (commercially obtained pooled human serum from Innovative Research, Inc. (Novi, MI, USA)) run every 10 samples and at the beginning and end of each batch of samples; (b) blinded, pooled study samples (5% for urine; 10% for serum) interspersed with the biological study samples (3 QCs/batch of 27 study samples) used to normalize batches of samples over the run; (c) 17 split-sample blinded duplicates of study samples also interspersed with study serum and urine samples, which were used to calculate reported median metabolite CV values; (d) isotope labeled internal standards for targeted analysis of aqueous metabolite (*n* = 33) and lipids (*n* = 54) in serum, which enabled absolute concentration determination and ensured evaluation of instrument stability and data quality; (e) internal standard, TSP, used to assess the spectral quality, calibrate spectra, and help with data normalization of urine NMR spectra; and (f) FAME (fatty acid methyl esters) of different fatty acid chain lengths for retention time indexing and myristic acid-d_27_ for help with metabolite identification and data normalization, respectively. Median CVs of blinded pooled study QC samples for the 4 different platforms (2 for serum analysis and 2 for urine analysis) across the samples were 4.4% for global NMR from 24 h urine, 5.6% for targeted lipidomics, 7.2% for targeted LC-MS/MS, and 21.6% for global GC-MS platforms.

### 2.5. Data Preprocessing and Analysis

Metabolite data from multiple high-dimensional platforms, including NMR, GC-MS, LC-MS, and lipidomics from both fasting serum and 24 h urine were utilized for this analysis. For metabolomic variables, those with more than 20% missing values were removed to ensure robust results. For the remaining variables, half of the minimum positive value was used to impute the values to approximate the detection limits. Normalization was performed for LC-MS and GC-MS data using local polynomial regression fitting (loess) over run order with the span parameter set at 0.75 within each batch among QC samples. All metabolites were log transformed using the natural logarithm to improve the normality of distributions prior to analysis. No transformations were conducted on covariates. Relative concentration data were used for the targeted aqueous LC-MS data. For ease of interpretation, unidentified metabolites from GC-MS were removed from the analysis. In total, 1108 metabolites were quantitated: *n* = 157 LC-MS aqueous metabolites and *n* = 756 LC-MS lipid species from 13 lipid classes were measured in serum; *n* = 58 NMR and *n* = 137 GC-MS were measured in urine. All metabolites included in analyses are listed in Supplementary Table S1.

We evaluated the pairwise correlations between these log-transformed metabolites and 29 covariates using Pearson’s correlation for continuous variables and Spearman Rank correlations for categorical variables. Covariates available and hypothesized to have the potential to affect metabolite concentrations included demographic variables: age (years; continuous), body mass index [BMI (kg/m)^2^; continuous], education (≤high school/GED; some college; ≥college degree; categorical); income (<USD 50 K versus ≥USD 50 K); marital status (never married/divorced or separated/widowed versus presently married/in a marriage-like relationship); self-reported race (Black compared to non-Hispanic white; there were insufficient observations for other race categories) and ethnicity (Hispanic compared to non-Hispanic whites; analyzed per categories at the time of data collection, which did not include an additional option for race within ethnicity) [48]; diet and lifestyle factors: total self-reported energy intake from FFQ (kcal/d; continuous); physical activity [total energy expended from recreational physical activity in metabolic equivalents (MET)/week; continuous]; current smoker (yes/no); alcohol intake (drinks/week; continuous); dietary supplement use [40]: multivitamin (yes/no); multivitamin plus mineral (yes/no); stress formula (yes/no); other combination pills (non-stress/multi; yes/no); any combination pills (yes/no); single ingredient supplements (yes/no); note that all participants reported taking at least one supplement; prescription medications (medications evaluated if used by ≥5% of study sample; yes/no): angiotensin-converting enzyme (ACE) inhibitors (also includes ACE inhibitors plus thiazide/thiazide-like agents and ACE inhibitor plus calcium channel blocker combinations); angiotensin II receptor antagonists (also includes angiotensin II plus thiazides); beta blockers (cardio-selective); bisphosphonates; calcium channel blockers; diuretic combinations (also includes thiazide-like diuretics); 3-hydroxy-3-methyl-glutaryl-coenzyme A (HMG-CoA) reductase inhibitors; nonsteroidal anti-inflammatory drugs (NSAIDS; also includes salicylates and Cox-2 inhibitors); proton pump inhibitors (also includes H-2 antagonists); and thyroid hormones; and other: season of FFQ completion (spring: March–May, summer: June–August; fall: September–November; winter: December–February; categorical), and sample storage time (years; continuous). Exclusions were made for extreme BMI (>50 kg/m^2^; *n* = 3) and biologically implausible energy intakes (<600 kcal/d; *n* = 6). These exclusions only affected analyses for these variables. Data were missing for the following variables: dietary supplement use *n* = 1; alcohol intake *n* = 7; education *n* = 3; current smoking status *n* = 10; and self-reported physical activity *n* = 6. These observations were not included in the analyses for that variable. Data are given for all pairwise associations remaining significant after controlling for false discovery rate (FDR < 0.01) for all covariates across each of the 4 metabolite platforms using the Benjamini–Hochberg algorithm [49]. All analyses were performed in Stata (v17, College Station, TX, USA).

## 3. Results

Characteristics of the 444 women in the NPAAS ancillary study are given in Table 1. The majority of women were white, educated with higher incomes, and non-smoking, and all women reported the use of dietary supplements.

Pairwise correlations for metabolites and covariates that were significant at FDR < 0.01 for all platforms are given in Table 2, Table 3, Table 4 and Table 5. For the LC-MS platform, 9 variables significantly correlated with serum metabolites: age (*n* = 18 metabolites), BMI (*n* = 16), alcohol intake (*n* = 3), self-reported race (Black compared to non-Hispanic white; *n* = 12), self-reported ethnicity (non-Hispanic white compared to Hispanic; *n* = 3), multivitamin plus mineral use (*n* = 3), non-stress combination pills (*n* = 2), any combination supplements (*n* = 6), and single supplements (*n* = 2) (Table 2). Most metabolites showed positive correlations with these covariates, except for BMI, race, and ethnicity, for which about half were inversely related. Absolute correlation values were weak to moderate, ranging from 0.19–0.47.

When we examined correlations between covariates and the 13 serum lipid classes, we found 5 classes that were lower among Black women compared to non-Hispanic white women: LPC, LPE, PC, DAG, and TAG. LPC and LPE classes were lower, and DAG and TAG classes were higher (correlated positively) with BMI. Alcohol intake was associated with higher PC and remained highly significant in sensitivity analyses restricted to women consuming <10 drinks/week. LPC as a class was higher with both non-stress supplement combinations and any combination supplement use (Table 3). Some individual lipid species were correlated with covariates; however, there were no specific patterns, suggesting minor shifts within lipid classes (Supplementary Table S2). Absolute correlations ranged from 0.18–0.36.

For the NMR platform, urinary metabolite concentrations were higher with increasing alcohol intake (*n* = 27), lower among Blacks compared to non-Hispanic whites (*n* = 42) and non-Hispanic whites compared to Hispanic/Latinas (*n* = 2), and higher with longer sample storage time (*n* = 15). Only one and two metabolites, respectively, were associated with BMI and age. Absolute correlations ranged from 0.18–0.58 (Table 4).

Similarly for GC-MS, urinary metabolite correlations were higher with increasing alcohol intake (*n* = 17) and sample storage time (*n* = 3), and lower among Blacks compared to non-Hispanic whites (*n* = 5). Absolute correlations ranged from 0.19–0.37 (Table 5).

## 4. Discussion

Clinical, demographic, and environmental factors may influence the metabolome separately from the main effect parameters of interest under investigations. This is particularly true for putative disease association biomarkers, which have often failed in validation studies due to the influences of clinical and demographic factors on metabolite levels and disease outcomes of interest. Our aim was to specifically determine which of the clinical, demographic, and lifestyle factors collected from an extensive list were highly correlated with metabolites to inform potential confounding factor selection, thereby improving model precision and reducing false discovery in future assessments of metabolite-disease associations. In this well-characterized observational cohort of 444 post-menopausal women with extensive demographic, clinical, and dietary data, we evaluated the correlation patterns of 1108 metabolites across 4 platforms with 29 covariates and observed significant, albeit modest, correlations for age, BMI, alcohol intake, and race. Dietary supplements and sample storage time were important for a smaller number of metabolites. Surprisingly, medication use had little significant impact on any metabolites, even with a high proportion (42%) of our study population taking at least one of the prescription medications evaluated.

Despite the influence of confounding factors on metabolite levels, efforts to identify these factors and quantify their contribution to affecting specific metabolite–outcome relations of interest have been limited. The exception is the assessment of well-known confounders such as age, BMI, and sex [2]. The impact of aging on the metabolome has been outlined in two recent reviews [50,51], which report altered metabolites in metabolic pathways primarily related to redox homeostasis, energy, and amino acid metabolism. In agreement with these reviews, we found that age was associated with many metabolites related to oxidative stress and amino acids. Similarly, comparisons of metabolite profiles across the continuum of BMI show large variations [52] with more profound associations on the metabolome demonstrated between lean and obese individuals [33]. We found that BMI was strongly associated with metabolites related to branched-chain amino acid and energy metabolism and several classes of lipids, including LPCs, LPEs, DAGs, and TAGs. Although our cohort comprised only women, other studies have reported the impacts of biological sex on amino acids, lipids, sugars, and keto acids (reviewed in [32]). Finally, several studies have also investigated circadian rhythms on metabolite levels; however, all samples in our cohort were obtained in the morning after an overnight fast, precluding this evaluation [53,54,55].

The finding that alcohol was highly correlated with many metabolites, particularly fatty acids, was not unexpected. Other investigators have evaluated the effects of alcohol on the blood and urine metabolome and have reported similar differences in lipids, [56,57,58] including higher diacyl PCs as we observed here. Although not significant at a class level, we also noted a shift (some higher, some lower) in numerous individual TAG species. Alterations in fat metabolism with alcohol intake are well recognized. [59] Mechanistically, alcohol promotes the accumulation of fat in the liver primarily through the substitution of ethanol instead of fatty acids for energy [60]. These fatty acids are then esterified into triglycerides, phospholipids, and cholesterol esters, which are secreted into circulation via lipoproteins [60]. This factor should be recorded if at all possible, particularly when utilizing lipidomics platforms. We should note that the women in this study were either non-drinkers or consumed only moderate amounts of alcohol (median = 0.4, with only 26 women reporting 10 or more drinks/week). In sensitivity analyses restricted to women consuming <10 drinks/week, associations were attenuated but did not disappear.

Many metabolites were also associated with self-identified race. Comparisons of Blacks and non-Hispanic whites revealed correlations with 5 classes of lipids, LPCs, LPEs, PCs, DAGs, and TAGs, and 42 aqueous metabolites, including TCA cycle intermediates and several amino acids, measured by NMR. Notably, all but a few significant lipids and urine metabolites were lower among Black women compared to non-Hispanic white women. We hypothesized that these relationships may be confounded by age, BMI or lipid-lowering medication use, as there were a greater proportion of Black women who were taking HMG-CoA reductase inhibitors relative to non-Hispanic white women (31% compared to 19%). However, the majority of significant correlations persisted with adjustment for any of these factors (data not shown). Most studies to date have evaluated the association of race and metabolites in the context of disease outcomes, making comparisons with our results difficult. Furthermore, the contribution of other confounding factors, such as adiposity (as opposed to BMI) in race–metabolite relationships may be difficult to separate.

Few studies have evaluated the contribution of multiple lifestyle and clinical factors to metabolite measures. We found no statistically significant correlations between the serum or urine metabolome and self-reported physical activity or energy intake. While self-report instruments such as FFQs or other questionnaires may be useful for capturing overall trends, i.e., in habitual dietary intakes or characterizing dietary or exercise patterns, they are subject to a lack of precision and systematic bias, especially for energy intake [61,62,63]. There were also no associations with metabolites for marital status, income, or education. Lastly, four of the six classes of dietary supplements were significantly correlated with vitamin metabolites, including pantothenate and pyridoxic acid, both B vitamins, likely reflecting high supplement use in this cohort. Of note, 373 of the 444 (84%) women in this study reported taking at least one supplement evaluated.

Factors related to sample handling, including processing and storage, can affect data quality owing to the instability of some metabolites or enzymatic activity during the clotting process. Some compounds, i.e., fatty acids and sulfur-containing amino acids, are more prone to deterioration than others. Most studies evaluating metabolite stability have focused on pre-analytic factors, short-term stability, or temperature. In one study evaluating the effects of long-term sample storage on plasma metabolite abundances, only 2% of metabolites tested were altered in the first 7 years of storage, with up to 26% after 16 years [64]. Metabolites that were most affected included complex lipids, fatty acids, and amino acids. Our samples were stored at −80 °C for an average of 11 years (range of 8.9–13.6) [38]. Metabolites most affected in our study were urinary amino acids and compounds involved in energy metabolism assayed by NMR, with all having higher concentrations with longer storage time, as has been noted in previous studies [65,66]. These observations likely reflect the high precision of NMR. Given that relatively few metabolites were altered overall, i.e., 18 of >1000, these data suggest that the majority of metabolites are stable with longer-term storage of ~11 years.

Many investigators have applied various statistical techniques in an effort to account for the effects of potential confounders. For example, Chen et al. used seemingly unrelated regression (SUR) to analyze metabolomics data from a colorectal cancer study and found that factors such as sex, BMI, age, alcohol use, and smoking had significant confounding effects, which could be minimized through appropriate modeling [31]. In our previous work focused on other NPAAS-related analyses, we have considered potential confounding factors for biomarker calibration–equation development to correct self-reported dietary intake [41,67,68]. We have found that including participant characteristics, such as BMI, age, dietary supplement use, the season of participation, and self-reported physical activity, in models with metabolites, strengthened diet–disease associations in larger WHI cohorts from which NPAAS derived [68,69]. Importantly, variables that have previously been found to be potential confounders between dietary exposures and chronic disease outcomes need to be adjusted in the disease risk models, even if they were part of the calibration equation development. While these studies illustrate the importance of various statistical techniques to alleviate confounding overall, none have quantified correlations between individual metabolites and potential confounders.

Strengths of this study include the evaluation of a large number of potential influencers on the metabolome, the use of multiple platforms with broad metabolome coverage to analyze both serum and urine, and a large, well-characterized post-menopausal cohort. We included many factors that have not been previously examined (e.g., dietary supplements, medication use, and long-term sample storage) and focused our analysis specifically on determining their effects on the metabolome. Nonetheless, there are some notable limitations. Importantly, our study sample included only post-menopausal women. Therefore, our results may not be generalizable to other populations. The use of boric acid as a preservative in urine may have affected some metabolite levels; however, we would expect that these would be the same across all samples. Although most factors considered had a wide range of variations and sufficient observations, some groups or factors had too few observations to provide meaningful correlations. For example, only 2% of women were current smokers. Additionally, we are evaluating these potential factors in isolation, whereas many may interact such that their individual contributions are hard to tease apart, e.g., adiposity may be a source of variation in the relationship between some covariates and many metabolites. Plans are underway to further these explorations with mathematical modeling and subsequent application to diet–disease risk assessments in other WHI cohorts.

In summary, of the factors evaluated, age, BMI, alcohol intake, and race had the largest impact on the serum metabolome, with most correlations ranging between 0.2 and 0.4. Dietary supplements and sample storage time had effects on a smaller subset of metabolites. Some factors, including commonly used prescription medications, marital status, income, education, and physical activity had little or no association with any metabolites in this cohort. The inclusion of the most relevant potential confounding factors, as well as the use of good methods to capture these data accurately, should be considered in biomarker-disease association studies using metabolomics to improve the reliability of metabolite-disease association analyses.

## Figures and Tables

**Table 1:** Covariates evaluated for the 444 women in the WHI NPAAS-OS observational cohort.

Variable ^a^	Mean ± SD or *n* (%) ^b^
Demographic	
Age (years)	69.0 (6.1)
Body mass index (kg/m^2^)	28.2 (6.0)
<25	156 (35%)
25–29	120 (27%)
≥30	171 (38%)
Education	
≤High school	64 (14%)
Some college	155 (36%)
≥College degree	222 (50%)
Income	
<USD 50 K/y	42 (10%)
≥USD 50 K/y	402 (90%)
Married	285 (64%)
Race ^c^	
White	315 (71%)
Black	79 (18%)
Asian	<10 (<1%)
American Indian/Alaskan Native	<10 (<1%)
More than one race	<10 (<1%)
Unknown/not reported	<10 (<1%)
Ethnicity	
Hispanic/Latina	68 (15%)
Diet and lifestyle	
Energy intake (kcal/d)	1619 (608)
Alcohol (drinks/week)	2.3 (4.3)
Current smoker	11 (2%)
Physical activity (MET h/week)	16.9 (range 0–85)
Dietary supplements ^d^	
Multivitamin	9 (2%)
Multivitamin plus minerals	249 (66%)
Stress multi-supplement	21 (5%)
Other supplement mixture (non-stress/multi)	255 (57%)
Any combination pills	347 (78%)
Single-ingredient supplements	226 (51%)
Medications	
ACE inhibitors	40 (9%)
Angiotensin receptor agonists	24 (5%)
Betablockers	38 (9%)
Bisphosphonates	38 (9%)
Calcium channel blockers	45 (10%)
Diuretic combinations/thiazides	60 (14%)
HMG CoA reductase inhibitors	83 (19%)
Non-steroidal anti-inflammatory drugs	30 (7%)
Proton pump inhibitors	36 (8%)
Thyroid hormones	56 (13%)
Other covariates	
Sample storage time (years)	11.3 (1.1)
Season of FFQ completion	
Fall	116 (26%)
Winter	120 (27%)
Spring	126 (28%)
Summer	82 (19%)

^a^ Age, body mass index, physical activity, season of participation, current smoking status, alcohol, dietary supplement use, and self-reported energy intake measures were taken at NPAAS clinic visits; medication use was taken at the end of the first extension. All other measures were obtained at enrollment in the Women’s Health Initiative (race and ethnicity; education; marital status; annual income). ^b^ Percents may not total 100 due to rounding or missingness (missing: dietary supplements *n* = 1; alcohol intake *n* = 7; education *n* = 3; current smoking status *n* = 10; physical activity *n* = 6). ^c^ Races reported per National Institutes of Health guidelines; however, data were analyzed per original collection for which racial and ethnic characterization was limited. ^d^ All women reported taking at least one dietary supplement. METs: metabolic equivalent hours per week of recreational physical activity.

**Table 2:** Variables correlated with metabolites at FDR < 0.01 by LC-MS, descending in level of statistical significance within each covariate.

	LCMS Serum			
Covariate	Metabolite	Rho ^a^	*p*-Value	FDR ^b^
Age (years; continuous)	Pseudouridine	0.37	1.20 × 10^−15^	1.95 × 10^−13^
	Arabitol/Xylitol	0.34	1.60 × 10^−13^	2.57 × 10^−11^
	N2,N2, Dimethylguanosine	0.33	2.20 × 10^−12^	3.38 × 10^−10^
	Inositol	0.27	7.00 × 10^−9^	1.07 × 10^−6^
	7-Methylguanine	0.27	7.30 × 10^−9^	1.11 × 10^−6^
	Homocitrulline	0.26	2.20 × 10^−8^	3.27 × 10^−6^
	5-Hydroxyl-indole-3-acetic acid	0.26	3.60 × 10^−8^	5.50 × 10^−6^
	N6, Acetyllysine	0.25	9.50 × 10^−8^	1.43 × 10^−5^
	Glucuronate	0.25	1.40 × 10^−7^	2. × 10^−5^
	N-formylmethionine	0.24	1.80 × 10^−7^	2.63 × 10^−5^
	2-Oxoisocaproic acid	−0.23	5.80 × 10^−7^	8.45 × 10^−5^
	N-Acetylneuraminate	0.23	6.20 × 10^−7^	9.13 × 10^−5^
	Threonic/Erythronic acid	0.23	1.10 × 10^−6^	0.0002
	SAH	0.22	4.00 × 10^−6^	0.0006
	N-Acetylalanine	0.21	5.80 × 10^−6^	0.0008
	Orotate	0.20	2.40 × 10^−5^	0.003
	N-Carbamoylbalanine	0.20	2.40 × 10^−5^	0.005
	Phenylacetylglutamine	0.19	6.80 × 10^−5^	0.009
Body Mass Index (continuous)	Urate	0.41	2.10 × 10^−19^	3.22 × 10^−17^
	N-Acetylglycine	−0.29	3.50 × 10^−10^	5.49 × 10^−8^
	Glutamic acid	0.27	5.30 × 10^−9^	8.18 × 10^−7^
	Cortisol	−0.27	1.10 × 10^−8^	1.76 × 10^−6^
	Oxalacetate	−0.25	7.00 × 10^−8^	1.07 × 10^−5^
	Glycine	−0.25	8.00 × 10^−8^	1.21 × 10^−5^
	Pyruvate	0.24	3.10 × 10^−7^	4.74 × 10^−5^
	Tyrosine	0.24	4.90 × 10^−7^	7.32 × 10^−5^
	Isovalerylcarnitine	0.23	8.70 × 10^−7^	0.0001
	Asparagine	−0.23	1.30 × 10^−6^	0.0002
	Citrulline	−0.23	1.70 × 10^−6^	0.0003
	Guanidinoacetate	−0.21	8.80 × 10^−6^	0.001
	Proline	0.20	1.70 × 10^−5^	0.002
	Glucose	0.20	2.70 × 10^−5^	0.004
	4-Pyridoxic acid	−0.19	4.60 × 10^−5^	0.007
	Valine	0.19	5.20 × 10^−5^	0.007
Race (Blacks versus non-Hispanic whites)	Ornithine	−0.29	8.39 × 10^−9^	1.32 × 10^−6^
	Inosine	0.28	6.53 × 10^−8^	1.02 × 10^−5^
	Trigonelline	−0.28	7.10 × 10^−8^	1.1 × 10^−5^
	γ-Tocopherol	0.27	2.11 × 10^−7^	3.24 × 10^−5^
	Guanosine	0.26	3.72 × 10^−7^	5.69 × 10^−5^
	Pantothenate	−0.26	7.19 × 10^−7^	0.0001
	Taurine	−0.24	5.22 × 10^−6^	0.0008
	Hydroxyproline	0.23	6.16 × 10^−6^	0.0009
	Genistate	−0.21	4.83 × 10^−5^	0.007
	Creatinine	0.21	5.12 × 10^−5^	0.008
	N-Acetylglutamine	0.21	5.13 × 10^−5^	0.008
	Xanthosine	0.21	6.03 × 10^−5^	0.009
Ethnicity (Hispanic whites versus non-Hispanic whites)	Taurine	−0.23	1.56 × 10^−5^	0.002
	Chenodeoxycholate	0.23	1.89 × 10^−5^	0.003
	Threonic acid/Erythronic acid	−0.22	2.83 × 10^−5^	0.004
Alcohol (drinks/week; continuous)	2-Hydroxyisovaleric acid	0.33	1.30 × 10^−12^	2.04 × 10^−10^
	Aarabitol/Xylitol	0.22	2.75 × 10^−6^	0.0004
	Inositol	0.20	1.68 × 10^−5^	0.003
Multivitamin use (yes/no)	Pantothenate	0.47	1.87 × 10^−25^	2.93 × 10^−23^
	4-Pyridoxic acid	0.34	3.48 × 10^−13^	5.42 × 10^−11^
	Riboflavin	0.19	5.71 × 10^−5^	0.009
Non-stress combination supplement (yes/no)	Pantothenate	0.30	1.91 × 10^−10^	3.00 × 10^−8^
	4-Pyridoxic acid	0.28	3.35 × 10^−9^	5.23 × 10^−7^
Any combination supplement (yes/no)	Pantothenate	0.46	4.00 × 10^−24^	6.28 × 10^−22^
	4-Pyridoxic acid	0.40	2.20 × 10^−18^	3.43 × 10^−16^
	A-Tocopherol	0.20	1.48 × 10^−5^	0.002
	2-Hydroxyisovaleric acid	−0.20	1.83 × 10^−5^	0.003
	Proline	−0.19	4.23 × 10^−5^	0.006
	Leucic acid	−0.19	4.44 × 10^−5^	0.007
Single formulation supplement (yes/no)	4-Pyridoxic acid	0.21	9.99 × 10^−6^	0.002
	Pantothenate	0.19	6.03 × 10^−5^	0.009

^a^ Correlations between variables and log-transformed metabolites (Pearson for continuous variables; Spearman for binary variables). ^b^ False Discovery Rate computed with Benjamini–Hochberg algorithm.

**Table 3:** Variables correlated with lipids at FDR < 0.01 by lipidomics, descending in level of statistical significance within each covariate.

LIPID Serum Class Absolute Quantitation			
Class ^a^	Rho ^b^	*p*-Value	FDR ^c^
Body mass index (continuous)			
LPC (8/17)	−0.31	1.90 × 10^−11^	2.46 × 10^−11^
LPE (4/8)	−0.30	1.50 × 10^−10^	1.76 × 10^−9^
DAG (13/29)	0.27	1.40 × 10^−8^	1.56 × 10^−7^
TAG (258/487)	0.26	2.30 × 10^−8^	2.26 × 10^−7^
Alcohol (drinks/week)			
PC (8/68)	0.23	7.50 × 10^−7^	0.0006
Race (Black versus non-Hispanic white)			
LPC (10/17)	−0.36	6.21 × 10^−13^	8.07 × 10^−12^
LPE (4/8)	−0.32	6.64 × 10^−10^	7.97 × 10^−9^
PC (25/68)	−0.19	0.0003	0.004
DAG (7/29)	−0.18	0.0004	0.004
TAG (138/487)	−0.18	0.0006	0.005
Non-stress combination supplement (yes/no)			
LPC (1/17)	0.18	0.0001	0.003
Any combination supplement (yes/no)			
LPC (2/17)	0.19	0.0004	0.0005

^a^ Number of individual significant species/total number evaluated within the lipid class. ^b^ Correlations between variables and log-transformed metabolites (Pearson for continuous variables; Spearman for binary variables). ^c^ False Discovery Rate computed with Benjamini–Hochberg algorithm.

**Table 4:** Variables correlated with metabolites at FDR < 0.01 by NMR, descending in level of statistical significance within each covariate.

NMR 24 h Urine			
Metabolite	Rho ^a^	*p*-Value	FDR ^b^
Age (continuous)			
4-Hydroxyhippuric acid	0.24	4.60 × 10^−7^	3.06 × 10^−5^
Glucose	0.22	3.90 × 10^−6^	0.0003
Body Mass Index (continuous)			
Fumarate	−0.19	0.0001	0.006
Alcohol (drinks/week; continuous)			
Ethyl alcohol	0.58	1.15 × 10^−39^	5.16 × 10^−46^
Propanediol	0.41	5.10 × 10^−10^	3.34 × 10^−17^
Fumarate	0.31	3.30 × 10^−11^	2.11 × 10^−9^
3-Hydroxyisovaleric acid	0.27	9.20 × 10^−9^	5.77 × 10^−7^
Succinic acid	0.26	2.80 × 10^−9^	1.72 × 10^−6^
Acetate	0.25	2.00 × 10^−7^	1.2 × 10^−5^
N-Methylnicotinic acid	0.24	7.20 × 10^−7^	4.32 × 10^−5^
Isobutyrate	0.23	1.70 × 10^−6^	0.0001
Proline	0.22	3.00 × 10^−6^	0.0002
Lactate	0.21	7.10 × 10^−6^	0.0004
Glycolate	0.21	7.80 × 10^−6^	0.0004
Tyrosine	0.21	9.10 × 10^−6^	0.0005
Hippurate	0.21	1.3 × 10^−5^	0.0007
Pseudouridine	0.21	1.40 × 10^−5^	0.0007
Threonine	0.20	1.9 × 10^−5^	0.001
2-Hydroxyisobutyrate	0.20	2.4 × 10^−5^	0.001
1-Methylhistidine	0.20	2.6 × 10^−5^	0.001
Valine	0.19	5.3 × 10^−5^	0.003
N-Methylnicotinamide	0.19	5.7 × 10^−5^	0.003
4-Hydroxyphenylacetate	0.19	6.6 × 10^−5^	0.003
Acetone	0.19	8.7 × 10^−5^	0.004
Glutamine	0.19	8.8 × 10^−5^	0.004
Guanidinoacetate	0.19	0.0001	0.005
Leucine	0.18	0.0002	0.009
Scylloinositol	0.18	0.0002	0.009
Sample storage time (years; continuous)			
Succinic acid	0.23	1.70 × 10^−6^	0.0001
Orotic acid	0.22	3.90 × 10^−6^	0.0003
Pseudouridine	0.21	1.4 × 10^−5^	0.0009
Tyrosine	0.20	1.8 × 10^−5^	0.001
Ascorbic acid	0.20	1.8 × 10^−5^	0.001
Fumarate	0.19	5.1 × 10^−5^	0.003
1-Methylhistidine	0.19	6.5 × 10^−5^	0.004
Proline	0.19	8.1 × 10^−5^	0.005
Threonine	0.19	8.6 × 10^−5^	0.005
Scylloinositol	0.19	9.4 × 10^−5^	0.005
Ureahippurate	0.18	0.0001	0.006
Acetone	0.18	0.0002	0.008
Methionine	0.18	0.0002	0.008
Valine	0.18	0.0002	0.009
Citrate	0.18	0.0002	0.009
Race (Blacks versus non-Hispanic whites)			
Fumarate	−0.37	4.89 × 10^−13^	3.23 × 10^−11^
1-Methylhistidine	−0.36	8.74 × 10^−13^	5.68 × 10^−11^
Succinicacid	−0.36	3.28 × 10^−12^	2.10 × 10^−10^
Glycolate	−0.34	3.60 × 10^−11^	2.27 × 10^−9^
Hippurate	−0.34	4.73 × 10^−11^	2.94 × 10^−9^
N-Methylnicotinicacid	−0.33	1.88 × 10^−10^	1.15 × 10^−8^
Proline	−0.29	1.19 × 10^−8^	7.16 × 10^−7^
Tyrosine	−0.29	3.05 × 10^−8^	1.77 × 10^−6^
Formate	−0.28	3.65 × 10^−8^	2.08 × 10^−6^
Acetate	−0.27	1.11 × 10^−7^	6.09 × 10^−6^
Glycine	−0.27	1.15 × 10^−7^	6.22 × 10^−6^
N-Methylnicotinamide	−0.27	1.32 × 10^−7^	7.01 × 10^−6^
Citrate	−0.27	1.47 × 10^−7^	7.63 × 10^−6^
Creatine	−0.27	1.80 × 10^−7^	9.16 × 10^−6^
Ethyl alcohol	−0.27	2.13 × 10^−7^	1.07 × 10^−5^
Glucose	−0.27	2.92 × 10^−7^	1.41 × 10^−5^
Allantoin	−0.27	2.94 × 10^−7^	1.41 × 10^−5^
Isobutyrate	−0.26	3.20 × 10^−7^	1.47 × 10^−5^
Glutamine	−0.26	4.58 × 10^−7^	2.06 × 10^−5^
Threonine	−0.26	7.26 × 10^−7^	3.2 × 10^−5^
Methionine	−0.26	7.83 × 10^−7^	3.37 × 10^−5^
Guanidinoacetate	−0.25	1.19 × 10^−6^	5.02 × 10^−5^
Ascorbic acid	−0.25	1.38 × 10^−6^	5.54 × 10^−5^
Valine	−0.25	1.54 × 10^−6^	6.0 × 10^−5^
Acetone	−0.25	2.18 × 10^−6^	8.27 × 10^−5^
Uracil	−0.24	3.27 × 10^−6^	0.0001
Leucine	−0.24	3.31 × 10^−6^	0.0001
2-Oxoglutarate	−0.24	3.79 × 10^−6^	0.0001
Pseudouridine	−0.23	7.12 × 10^−6^	0.0002
Alanine	−0.23	1.1 × 10^−5^	0.0004
Urea	−0.22	1.69 × 10^−5^	0.0005
Scylloinositol	−0.22	1.86 × 10^−5^	0.0006
Methylguanidine	−0.22	2.46 × 10^−5^	0.0007
3-Hydroxyisovaleric acid	−0.22	3.45 × 10^−5^	0.001
4-Hydroxyphenylacetate	−0.22	3.63 × 10^−5^	0.001
Acetoacetate	−0.21	4.12 × 10^−5^	0.001
Trimethylamine	−0.21	5.16 × 10^−5^	0.001
Isoleucine	−0.21	6.57 × 10^−5^	0.002
Histidine	−0.20	0.0001	0.003
Phosphocholine	−0.20	0.0001	0.003
Orotic acid	−0.19	0.0002	0.004
2-Hydroxyisobutyrate	−0.18	0.0005	0.009
Ethnicity (Hispanic whites vs. Non-Hispanic whites)			
Citrate	−0.21	7.01 × 10^−5^	0.005
Ascorbic acid	−0.21	7.76 × 10^−5^	0.005

^a^ Correlations between variables and log-transformed metabolites (Pearson for continuous variables; Spearman for binary variables). ^b^ False Discovery Rate computed with Benjamini–Hochberg algorithm.

**Table 5:** Variables correlated with metabolites at FDR < 0.01 by GC-MS, descending in level of statistical significance within each covariate.

GC-MS 24 h Urine			
Metabolite	Rho ^a^	*p*-Value	FDR ^b^
Alcohol (drinks/week; continuous)			
D-Galacturonic acid	0.37	8.20 × 10^−16^	8.77 × 10^−14^
L-Threonine	0.27	6.50 × 10^−9^	6.89 × 10^−7^
Methyl-3,4-dimethoxyphenyl hydroxy acetate	0.26	6.70 × 10^−8^	7.04 × 10^−6^
Decanoic acid	0.24	2.70 × 10^−7^	2.81 × 10^−5^
Indol-5-ol	0.24	5.10 × 10^−7^	5.25 × 10^−5^
D-Talose	0.23	1.10 × 10^−6^	0.0001
Methylglycocholate	0.21	6.80 × 10^−6^	0.0007
D-Psicose	0.21	1.3 × 10^−5^	0.001
Paracetamol	0.21	1.52 × 10^−5^	0.001
α-D-Glucopyranoside, methyl-2-(acetylamino)-2-deoxy- cyclicboronate	0.20	2.3 × 10^−5^	0.002
Levoglucosan	0.20	2.3 × 10^−5^	0.002
Dodecanoic acid	0.20	3.31 × 10^−5^	0.003
Tartaric acid	0.20	3.33 × 10^−5^	0.003
Quininic acid	0.20	3.5 × 10^−5^	0.003
D-Ribofuranose	0.20	3.8 × 10^−5^	0.004
Hexadecanoic acid	0.19	8.4 × 10^−5^	0.008
D-Glucose, 6-O-α-D- galactopyanosyl	0.19	0.0001	0.009
Sample storage time (years; continuous)			
Tartaric acid	0.21	1.0 × 10^−5^	0.001
Gabapentin-lactam	0.19	6.4 × 10^−5^	0.007
D-Talose	0.19	9.4 × 10^−5^	0.009
Race (Black versus non-Hispanic white)			
Pentanedioic acid	−0.24	2.20 × 10^−7^	2.35 × 10^−5^
Levoglucosan	−0.22	2.00 × 10^−6^	0.0002
a-D-Glucopyranoside, methyl-2-(acetylamino)-2-deoxy-3-O	−0.21	8.10 × 10^−6^	0.0009
Gabapentin-lactam	−0.20	2.1 × 10^−5^	0.00
D-Tagatose	−0.20	3.6 × 10^−5^	0.004

^a^ Correlations between variables and log-transformed metabolites (Pearson for continuous variables; Spearman for binary variables). ^b^ False Discovery Rate computed with Benjamini–Hochberg algorithm.

## Data Availability

Data, codebook, and analytic code used in this report may be accessed in a collaborative mode as described on the Women’s Health Initiative website (www.whi.org).

## Supplementary Materials

The following supporting information can be downloaded at: https://www.mdpi.com/article/10.3390/metabo13040514/s1, Table S1: All measured metabolites; Table S2: Significant lipids.
